# Factors Associated with Urban Risk-Taking Behaviour during 2018 Typhoon Mangkhut: A Cross Sectional Study

**DOI:** 10.3390/ijerph17114150

**Published:** 2020-06-10

**Authors:** Evan Su Wei Shang, Eugene Siu Kai Lo, Zhe Huang, Kevin Kei Ching Hung, Emily Ying Yang Chan

**Affiliations:** 1 Collaborating Centre for Oxford University and CUHK for Disaster and Medical Humanitarian Response (CCOUC), The Chinese University of Hong Kong, Hong Kong, China; evanshang98@link.cuhk.edu.hk (E.S.W.S.); euglsk@cuhk.edu.hk (E.S.K.L.); huangzhe@cuhk.edu.hk (Z.H.); kevin.hung@cuhk.edu.hk (K.K.C.H.); 2 JC School of Public Health and Primary Care, The Chinese University of Hong Kong, Hong Kong, China; 3 Accident & Emergency Medicine Academic Unit, The Chinese University of Hong Kong, Prince of Wales Hospital, Hong Kong, China; 4 Nuffield Department of Medicine, University of Oxford, Oxford OX37BN, UK

**Keywords:** typhoon, hurricane, cyclone, strong wind levels, natural disaster, Health-EDRM, urban, risk-taking behaviour, sensation seeking

## Abstract

Although much of the health emergency and disaster risk management (Health-EDRM) literature evaluates methods to protect health assets and mitigate health risks from disasters, there is a lack of research into those who have taken high-risk behaviour during extreme events. The study’s main objective is to examine the association between engaging in high-risk behaviour and factors including sociodemographic characteristics, disaster risk perception and household preparedness during a super typhoon. A computerized randomized digit dialling cross-sectional household survey was conducted in Hong Kong, an urban metropolis, two weeks after the landing of Typhoon Mangkhut. Telephone interviews were conducted in Cantonese with adult residents. The response rate was 23.8% and the sample was representative of the Hong Kong population. Multivariable logistic regressions of 521 respondents adjusted with age and gender found education, income, risk perception and disaster preparedness were insignificantly associated with risk-taking behaviour during typhoons. This suggests that other factors may be involved in driving this behaviour, such as a general tendency to underestimate risk or sensation seeking. Further Health-EDRM research into risk-taking and sensation seeking behaviour during extreme events is needed to identify policy measures.

## 1. Introduction

Asia is particularly at risk of tropical cyclones, also known as typhoons in the western Pacific, with around half of worldwide tropical cyclones recorded and more than 90% of cyclone-related deaths being from this region [1]. Typhoon Mangkhut, the fourth supertyphoon in the 2018 Pacific typhoon season, started east of Guam in September 2018 and caused devastating damage to the Philippines [2] and South China [3]. In Hong Kong, tropical cyclone warning signal No. 10 (the highest signal for Hong Kong) was hoisted for 10 h, with the highest wind speed exceeding 150 km/h, just lower than the respective records held by Typhoon York in 1999 and Typhoon Ellen in 1983 [4]. Although there were no deaths reported directly due to the typhoon, more than 60,000 trees had reportedly fallen and around 13,500 households experienced a power outage for more than 24 h in Hong Kong [5]. Record breaking storm surges coupled with high waves caused flooding in various coastal areas, despite not being at high tide, with hundreds of stranded or damaged vessels. These occurrences highlighted the increasing environmental hazards and potential health risks faced by coastal communities around the world as typhoons become more frequent and severe through climate change [6].

Health emergency and disaster risk management (Health-EDRM) is a field which involves the systematic analysis and management of health risks surrounding emergencies and disasters. By reducing risk and vulnerabilities and improving preparedness, response and recovery measures, the impact of disasters can be minimised [7]. Health-EDRM focuses on increasing the resilience of individuals, households and communities through education interventions, promotion of disaster risk reduction and supporting mechanisms in place to mitigate impacts of disasters [8]. These can include accessible disaster warning and information, protection of key health services and securing the basic needs of the population. By understanding the causes and factors in play regarding disaster risk, Health-EDRM provides evidence to drive future interventions and policy decisions.

A previous study on urban disaster preparedness in Hong Kong discovered that only 20.6% of respondents chose the correct action to take while major disaster warnings were in force (such as staying in a safe place until heavy rain has passed) [9]. However, few studies investigate individuals intentionally performing risk-taking behaviour during natural disasters. Research on ‘storm chasers’ (those who intercept severe convective storms for sport or for scientific research) [10] in the United States has examined individual perception of recreational storm chasing to dispel its myths [11], operational methods of storm chasing tour groups [12] and sensation seeking traits associated with tour participants [13]. There is a lack of research into such behaviour outside of the United States, especially in urban areas directly impacted by meteorological disasters. Various studies by Zuckerman on sensation seeking have identified its dimensions [14] and relationships to different aspects through the sensation seeking scale (SSS) [15]. A meta-analysis into sex differences in sensation seeking showed that men scored higher than women using Zuckerman’s SSS-V and could be explained by evolutionary psychology and through a cultural socialisation perspective [16]. Furthermore, a review of behavioural and biological correlates of sensation seeking also found males significantly outscored females on total sensation seeking in different western countries and sensation seeking typically decreases with increasing age after adolescence [17]. Education and occupation were less associated with sensation seeking, particularly for females. The review also highlighted high sensation seekers perceived risks in the environment as less threatening compared to low sensation seekers and did not perceive engaging in high-risk behaviours would lead to negative consequences.

This article is an extension of the cross-sectional study investigating risk perception, household preparedness, and self-reported short-term impacts of typhoons after Typhoon Mangkhut [18]. Our previous published paper highlighted 16.0% of respondents reportedly left their homes when the warning signal was T8 or above, when the typhoon was at the height of strength. This behaviour will henceforth be referred to as risk-taking behaviour during typhoons (RBDT). Out of those respondents, a majority (74.7%) performed RBDT for non-essential reasons. The previous article also found that men and younger respondents were more likely to execute non-essential RBDT. Using the same dataset, the objectives of the current study are to expand on these findings and investigate other factors that may be related to RBDT, namely (1) to identify the sociodemographic characteristics of those who left their homes when the storm was at its height of strength (i.e., the warning signal was T8 or above); and (2) to explore the associations between sociodemographic factors other than age and gender, risk perception, household preparedness, and RBDT for non-essential purposes. The study findings will offer further insight into risk-taking behaviour during natural disasters to guide future interventions and policy on preventing such unnecessary high-risk behaviour.

## 2. Materials and Methods

**Figure 1:**
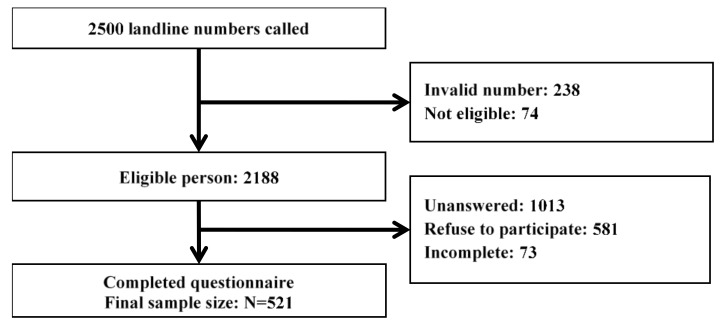
The recruitment details in the telephone survey.

A computerized digit dialling population-based household telephone survey was conducted from 17 September 2018 to 2 October 2018, right after the date of Typhoon Mangkhut landing in Hong Kong. Random digit dialling and the last birthday method [19] (interviewer would seek the household member whose birthday was the closest to the interview date) were used to ensure randomization in the study. Hong Kong residents who understood Cantonese and were 18 years old or older were interviewed. In total, 2500 landline numbers were called, and 521 respondents were successfully recruited (Figure 1). The response rate was 23.8% (response rate: 521 (final sample size) /2188 (eligible persons)). Please refer to the previous published study for study design [18].

This study investigates the associations between non-essential RBDT and the sociodemographic factors of education and income; indicators of risk perception including perception of Hong Kong being susceptible to disasters, perception of the impact of Typhoon Mangkhut compared to expectations and concern for the safety of oneself and family members; indicators of household preparedness including food and water reserves prepared routinely or specifically for Typhoon Mangkhut.

This paper considers the act of going outside during the strongest typhoon winds as risk-taking (RBDT) or high-risk behaviour, regardless of the reasoning. Those who go outside for work or emergency related purposes would be considered as engaging in socially acceptable or understandable risk-taking behaviour. This article focuses on active risk-taking behaviour, where individuals remove themselves from areas of safety to head into areas posing health risks, and does not investigate passive risk-taking behaviour, such as failure to act, evacuate or engage in other safety-seeking behaviour. This is because there are potentially different motivating factors, rationale and mechanisms involved in these two types of behaviour and research has found that passive risks are associated with a lower risk perception than equivalent active risks [20]. Previous literature has investigated passive risks associated with disasters and relevant methods to protect passive risk, but there is a lack of research into active risk-taking behaviour.

To clarify, the respondents were asked whether they left their homes to go outside during Typhoon Mangkhut while the typhoon warning signal T8 or above was in force. Those who left their home to address any perceived urgent and unexpected situations that required immediate action to prevent further deterioration, such as “due to injury or disease”, were classified as having ‘emergency’ reasons. Those who did not have to manage such pressing issues or work-related duties but left their home for other reasons, such as “eating a meal or watching a movie”, were categorized as having ‘non-emergency’ reasons. This paper will hence refer to RBDT due to ‘non-emergency’, also referred to as non-emergency and non-occupational reasons in our previous published paper, as non-essential reasons. Verbal informed consent was obtained at the beginning of the interview. The ethical approval of this study was obtained from the Survey Behavioural Research Committee at the Chinese University of Hong Kong (SBRE-18-075).

Descriptive chi-square (or X^2^) tests were used to compare the study population and the respondents who reported going outdoors during Typhoon Mangkhut. We conducted univariate analyses to identify associations between sociodemographic characteristics, risk perception, disaster preparedness factors and the risk-taking behaviour. Multivariable logistic regression was performed to identify factors related to going outdoors during strong typhoon winds for ‘non-emergency’ reasons, using variables with at least marginal statistical association in the univariate analysis (*p* < 0.10). Age and gender were covariates for the multivariable model base. All odds ratios (OR) present in this paper were adjusted odds ratios from the multivariable models. Statistical analyses were performed using IBM SPSS 24 (International Business Machines Corporation, Armonk, NY, USA) [21] and statistical significance was set at α = 0.05 two-sided.

## 3. Results

Data were collected from 17 September 2018 to 2 October 2018. The final sample size constituted 521 valid respondents (the response rate was 23.8% among eligible people called). The study population were comparable with the Hong Kong 2016 census data, except the study population were proportionally more middle age (age 45–64), more at the post-secondary education level and had higher income. Please refer to the previous paper [18] for further information and detailed analyses.

### 3.1. Description of the Study Population

For the descriptive comparison (Table 1), it was found that men were more likely to engage in non-essential RBDT (*p* = 0.006). There were no significant associations between RBDT and marital status, income, and respondents with chronic disease. Of the respondents with occupations which require emergency work while typhoon No. 8 or higher was in force, 72.2% reported staying home during Typhoon Mangkhut when the typhoon was at the height of strength. In addition, respondents who had occupations relating to “Sales and services” and “Elementary occupation” were more likely to leave their homes for emergency or work reasons. Respondents who participated in RBDT for emergency or work reasons were found to be more likely to obtain their weather-related information during this typhoon through television and newspapers and less likely through websites and mobile apps.

### 3.2. Factors Associated with Non-Essential RBDT

In the multivariable logistic regressions (Table 2), the relationship between various sociodemographic details, the subject’s perception and preparedness for the typhoon and non-essential RBDT was examined. As reported in the previous article, being male and younger were found to have higher odds in performing non-essential RBDT than those that did not go outside. All factors investigated in this article such as education, disaster risk perception or household preparedness were not found to have a statistically significant association with non-essential RBDT after adjusting for age and gender. Food and water reserves (both routine and specifically prepared for Typhoon Mangkhut) were also not found to be related.

## 4. Discussion

The current study is an extension of the previous published paper [18], which investigated risk perception, household preparedness, and self-reported short-term impacts of typhoons after Typhoon Mangkhut. This study aims to identify the sociodemographic characteristics of those who performed RBDT and investigate correlations between other sociodemographic characteristics apart from age and gender, risk perception and preparedness, and non-essential RBDT. There were no significant associations between RBDT and marital status, income, and respondents with chronic disease. Respondents who participated in RBDT for emergency or work reasons were more likely to watch television and read newspapers to obtain their weather-related information and less likely through websites and mobile apps. Education, income, risk perception and preparedness were found to be insignificantly associated with non-essential RBDT.

### 4.1. Comparison between Household Preparedness and Risk-Taking Behaviour

Household preparedness and risk-taking behaviour may be negatively associated, as household preparedness represents active protective behaviour, while non-essential RBDT involves potentially injurious active behaviour. Although this study did not find any significant negative association between higher educational level, higher risk perception and routine emergency preparedness with non-essential RBDT, the previous published paper [18] found a positive association of these factors with individuals who engaged in household preparedness. The results suggest these two behaviours may have different perceived levels of risk and/or involve separate rationale, such as respondents not considering non-essential RBDT as high-risk activity but performing household preparedness to ensure adequate supplies for family members. However, there is a lack of data in this study on the magnitude and severity of risk perceived from the typhoon to support this hypothesis. In addition, this study could not indirectly gauge the relative amount of risk respondents were willing to take for RBDT, which could be independent from perception of general risks of the typhoon and disasters.

### 4.2. Difference between Typhoon Risk Perception and Risk Perception of Non-Essential RBDT

Although there was no association between perceived impact of Typhoon Mangkhut compared to expectations and non-essential RBDT, 80.9% of all respondents thought the impact of the typhoon was similar or less than expected. This may suggest that fewer people underestimate the risks of typhoons, even after the recent typhoon influence, given that Typhoon Mangkhut was objectively one of the strongest typhoons in Hong Kong to date. The literature on sensation seeking involving demographics [17] has also shown that high sensation seekers have lower risk perception of activities they have not engaged in before and are less likely to perceive the negative consequences of risk-taking behaviour. Therefore, there may be a difference between the perceived risk of typhoons or the resulting impacts and non-essential RBDT which is an active behaviour. High sensation seekers may identify typhoons as events that cause harm but do not perceive the negative consequences of non-essential RBDT. As there is a lack of data directly investigating the reasons people performed non-essential RBDT, this study explores other inferred and documented possible rationale.

### 4.3. Other Reasons for Non-Essential RBDT

This study found food and potable water reserves, whether prepared regularly or specifically for this typhoon, were not associated with respondents going outside for non-essential reasons at the height of the storm. In addition to being markers of household preparedness, the reserves and the lack thereof may indicate situations of non-essential character, which could motivate individuals to leave their homes under the rationale of necessity. The results suggest that seeking these two necessities were not primary reasons for non-essential RBDT.

News outlets have documented a case of an elderly man in Hong Kong stranded at sea and requiring rescue after swimming during Typhoon Haima while the typhoon signal No. 8 was in force [22], highlighting an instance of RBDT which would have caused significant harm if rescue operations did not take place. As the man’s motives were not interviewed and reported, the behaviour could have been due to an underestimation of the risk involved or due to sensation seeking. Despite warnings from the Hong Kong government [23] and Hong Kong Observatory [24] before and during Typhoon Mangkhut, people were reported participating in sensation seeking behaviour, such as ‘experiencing the wind’ and engaging in disaster photography [25,26]. High-risk behaviour during extreme weather has also been documented in the news, with 300 firefighters mobilised to rescue scores of ‘frost chasers’ and other hikers from Hong Kong’s highest peak during the city’s coldest day in six decades [27]. Although the news reports did not interview the individuals and verify their desire to seek novel and intense experiences, it is clear that the risk-taking behaviours reported were performed directly to experience aspects of the disasters.

### 4.4. Ethical Concerns in Rescue Operations

There is also an ethical argument on the duty of firefighters who bear personal risk and danger to help those engaging in sensation seeking behaviour. A fireman in Hong Kong died in 2017 after sustaining a cliff fall while rescuing a pair of hikers [28], highlighting the risk and peril involved even in normal conditions. While rescuers have a duty to respond to emergencies, in cases involving sensation seeking behaviour, this creates a moral issue posing unnecessary risks to the rescuers in such extreme weather events. Rescue operations may also hinder other emergency responses and invoke issues of justice and equitable resource allocation. In total, 160 firemen were involved in a 24 h operation to rescue a pair of hikers who were stuck on a hiking trail during Tropical Storm Pakhar, which struck Hong Kong only days after Typhoon Hato. The entire operation included a total of ten ambulances and thirty-one fire engines, with an estimated total cost of more than 344,000 HKD in staffing costs alone [29].

### 4.5. What Is the Gap Found in This Study?

One of the major gaps found in this study is that education, disaster risk perception and disaster preparedness were not found to be associated with whether respondents engaged in unexplained risky behaviour. Common understanding would suggest that those with better understanding of the risks presented and especially those concerned about the safety of themselves and their family members during the typhoon would practice less risk-taking or sensation seeking. This suggests that knowledge-based interventions may not be as effective in deterring individuals from engaging in non-essential RBDT.

### 4.6. Recommendations

As younger aged males were found to be more likely to engage in non-essential RBDT, health promotion targeting this group may be more effective, possibly through official government websites/apps or well-placed advertisements in social media platforms. Legislation limiting accessibility to areas of higher risk during typhoons, such as waterfronts and beaches, may also have a beneficial effect on warding off risk-taking behaviour. Numerous provinces and cities in the Philippines have imposed liquor bans during typhoons to prevent inebriated individuals from hindering relief operations [30,31,32], but the effectiveness of such legislation have not been investigated. Furthermore, legal restrictions of outdoor movement in the interests of public health may be more socially acceptable due to the experiences of social distancing amid COVID-19.

As some respondents were found to engage in RBDT for work purposes, the updated “Code of Practice in Times of Typhoons and Rainstorms”, published by the Hong Kong Labour Department [33], is a crucial document in mitigating health risks to essential workers. Although the guidelines are not compulsory by law, employers and employees must discuss and clearly outline methods to protect the health of those working during extreme conditions. Interventions targeting this group may be more effective if broadcasted through television or newspapers. Thus, a multi-stakeholder approach should be adopted to promote work-related safety and reduce RBDT. This also has a global implication on preventable injury risk reduction, with international planning and discussions necessary in the near future before stronger natural disaster occur driven by climate change.

### 4.7. Limitations

The main limitation of the analysis is that the results do not demonstrate a causative effect since this study used descriptive analysis and logistic regression as a cross-sectional study. There may also be reporting bias as respondents may present with higher social desirability response bias due to telephone interviews [34]. In addition, analyses involving risk perception asked two weeks after may not correlate directly with risky actions performed during the disaster. This study is also unable to distinguish whether those who executed non-essential RBDT carried out high-risk behaviour due to perception of low-risk or for sensation seeking purposes. There may also be other reasons forcing individuals to remain outdoors during the typhoon, such as street sleepers unable to reach temporary shelters, unaccounted for due to the study design. Recruiting participants using the last birthday method also limits analysis on household preparedness as the recruited participant may not be the decision-maker for household preparedness [35], and thus, unfamiliar with the measures taken.

### 4.8. Future Research Directions

Future studies can directly investigate the reasons or rationale behind RBDT and explore the extent of such behaviour in detail. The sensation seeking trait and relevant factors could also be examined to determine its association and relevance to RBDT by using a modified version of the sensation seeking scale. Research into interpersonal perspectives through injunctive safety norms [36] and motivating factors for adaptation behaviour, such as descriptive norms [37], may also offer further insight into risk-taking behaviour during disasters. Future studies regarding disaster risk perception may also consider quantifying risk perception using balanced rating scales, as the degree of risk perceived is likely important when analysing risk-taking behaviour.

## 5. Conclusions

While age and gender were associated with risk-taking behaviour during natural disasters, other sociodemographic characteristics (such as education) and measures of disaster risk perception or disaster preparedness were not found to be correlated with non-essential RBDT. Despite the lack of literature investigating this phenomenon, media outlets in Hong Kong have reported several cases of sensation seeking behaviour during typhoons. Future studies are necessary to investigate the scope of risk-taking behaviour during disasters and the reasons driving this behaviour. Although there have been limited reports of injuries caused by risk-taking behaviour, relevant policy makers should begin to discuss and implement solutions to prevent any accidents and reduce potential extra burden on the emergency response system.

## Figures and Tables

**Table 1:** Descriptive table of the study population.

Characteristics		Go Outdoor for Emergency/Work Reasons (*n* = 21)	Go Outdoor for Non-Essential Reasons (*n* = 62)	Did Not Go Outdoor (*n* = 438)	*p*-Value
Gender	Male	9 (42.9%)	38 (61.3%)	174 (39.7%)	0.006
	Female	12 (57.1%)	24 (38.7%)	264 (60.3%)	
Age	18–24	2 (9.5%)	14 (22.6%)	47 (10.7%)	0.078
	25–44	9 (42.9%)	18 (29.0%)	127 (29.0%)	
	45–64	9 (42.9%)	24 (38.7%)	191 (43.6%)	
	≥65	1 (4.8%)	6 (9.7%)	73 (16.7%)	
Education attainment	Primary or below	2 (10.0%)	5 (8.1%)	49 (11.3%)	0.407
	Secondary	7 (35.0%)	18 (29.0%)	170 (39.2%)	
	Post-secondary	11 (55.0%)	39 (62.9%)	215 (49.5%)	
Marital status	Single	8 (38.1%)	28 (45.2%)	176 (40.2%)	0.734
	Married	13 (61.9%)	34 (54.8%)	262 (59.8%)	
Income	<2000–9999	0 (0.0%)	5 (8.9%)	40 (9.9%)	0.462
	10,000–19,999	2 (10.5%)	5 (8.9%)	66 (16.3%)	
	20,000–39,999	6 (31.6%)	21 (37.5%)	133 (32.9%)	
	≥40,000	11 (57.9%)	25 (44.6%)	165 (40.8%)	
Occupation	Manager/Professional/Clerk	9 (45.0%)	24 (41.4%)	151 (35.4%)	
	Sales & Services	5 (25.0%)	2 (3.4%)	35 (8.2%)	
	Craft related/ Machinery labour	0 (0.0%)	5 (8.6%)	20 (4.7%)	0.033
	Elementary occupation	3 (15.0%)	3 (5.2%)	23 (5.4%)	
	Housewives/Students	1 (5.0%)	14 (24.1%)	115 (26.9%)	
	Unemployed/Retired	2 (10.0%)	10 (17.2%)	83 (19.4%)	
Chronic disease	Yes	4 (19.0%)	10 (16.7%)	77 (17.7%)	0.965
	No	17 (81.0%)	50 (83.3%)	357 (82.3%)	
Routinely work during typhoon signal no.8	Yes	15 (83.3%)	4 (9.3%)	35 (13.4%)	<0.001
	No	3 (16.7%)	39 (90.7%)	227 (86.6%)	
Occupation involving mainly outdoor work	Yes	3 (17.6%)	10 (23.3%)	43 (16.8%)	0.591
	No	14 (82.4%)	33 (76.7%)	213 (83.2%)	
Channel of obtaining weather information	Television	12 (57.1%)	26 (41.9%)	236 (53.9%)	0.015
	Radio	0 (0.0%)	6 (9.7%)	24 (5.5%)	
	Website/Smartphone platform	6 (28.6%)	28 (45.2%)	168 (38.4%)	
	Newspaper or others	3 (14.3%)	2 (3.2%)	10 (2.3%)	
Disaster preparation immediately prior to the typhoon	No	0 (0.0%)	4 (6.5%)	28 (6.4%)	0.489
	Yes	21 (100.0%)	58 (93.5%)	410 (93.6%)	

**Table 2:** Chi-square comparison and multivariable logistic regressions of the associating factors towards non-essential RBDT.

Factors		χ^2^ Test			Logistic Regression	
		Stayed Indoor	Non-essential RBDT	*p*-Value	OR (95% CI)	*p*-Value
Gender ^a^	Male	174 (39.7%)	38 (61.3%)	0.001	Ref.	
	Female	264 (60.3%)	24 (38.7%)		0.435 (0.248–0.761)	0.004
Age ^a^	18–24	47 (10.7%)	14 (22.6%)	0.041	Ref.	
	25–44	127 (29.0%)	18 (29.0%)		0.573 (0.260–1.263)	0.167
	45–64	191 (43.6%)	24 (38.7%)		0.527 (0.248–1.118)	0.095
	≥65	73 (16.7%)	6 (9.7%)		0.297 (0.106–0.833)	0.021
Education attainment ^b^	Primary or below	49 (11.3%)	5 (8.1%)	0.144	Ref.	
	Secondary	170 (39.2%)	18 (29.0%)		0.799 (0.269–2.374)	0.687
	Post-secondary	215 (49.5%)	39 (62.9%)		1.110 (0.363–3.392)	0.854
Income ^b^	<2000–9999	40 (9.9%)	5 (8.9%)	0.517	Ref.	
	10,000–19,999	66 (16.3%)	5 (8.9%)		0.465 (0.120–1.796)	0.267
	20,000–39,999	133 (32.9%)	21 (37.5%)		0.897 (0.28–2.784)	0.851
	≥40,000	165 (40.98	25 (44.6%)		0.821 (0.264–2.555)	0.733
Perceived Hong Kong to be susceptible to disasters ^b^	No	35 (8.0%)	4 (6.5%)	0.669	Ref.	
	Yes	402 (92.0%)	58 (93.5%)		1.474 (0.494–4.398)	0.486
Perceived impact of Typhoon Mangkhut compared to expectations ^b^	Less than expected	118 (27.1%)	16 (25.8%)	0.919	Ref.	
	Same as expected	235 (54.0%)	33 (53.2%)		0.919 (0.477–1.773)	0.802
	Larger than expected	82 (18.9%)	13 (21.0%)		1.032 (0.462–2.303)	0.940
Concerned for the safety of oneself and family members ^b^	No	159 (36.3%)	17 (27.4%)	0.171	Ref.	
	Yes	279 (63.7%)	45 (72.6%)		1.585 (0.860–2.922)	0.140
Practiced disaster preparedness immediately prior to the typhoon ^b^	No	28 (6.4%)	4 (6.5%)	0.986	Ref.	
	Yes	410 (93.6%)	58 (93.5%)		1.077 (0.355–3.264)	0.896
Preparedness: routine food reserves ^b^	No	81 (18.5%)	7 (11.3%)	0.163	Ref.	
	Yes	357 (81.5%)	55 (88.7%)		1.663 (0.717–3.857)	0.236
Preparedness: routine potable water reserves ^b^	No	223 (50.9%)	32 (51.6%)	0.918	Ref.	
	Yes	215 (49.1%)	30 (48.4%)		0.842 (0.484–1.463)	0.541
Preparedness: food reserves specifically for Typhoon Mangkhut ^b^	No	137 (31.3%)	25 (40.3%)	0.154	Ref.	
	Yes	301 (68.7%)	37 (59.7%)		0.666 (0.378–1.174)	0.160
Preparedness: potable water reserves specifically for Typhoon Mangkhut ^b^	No	272 (62.1%)	39 (62.9%)	0.903	Ref.	
	Yes	166 (37.9%)	23 (37.1%)		0.894 (0.506–1.581)	0.700

^a^ is the multivariable regression of age and gender; ^b^ is the logistic regression adjusted with age and gender.
